# Infrared Spectral Classification of Natural Bitumens for Their Rheological and Thermophysical Characterization

**DOI:** 10.3390/molecules28052065

**Published:** 2023-02-22

**Authors:** Anastasiya Y. Yadykova, Larisa A. Strelets, Sergey O. Ilyin

**Affiliations:** 1 A.V. Topchiev Institute of Petrochemical Synthesis, Russian Academy of Sciences, 29 Leninsky Prospect, 119991 Moscow, Russia; 2 Institute of Petroleum Chemistry, Siberian Branch of Russian Academy of Sciences, 4 Academichesky Prospekt, 634055 Tomsk, Russia

**Keywords:** bitumen, maltha, asphalt, ozokerite, gilsonite, asphaltite, waxy oil, heavy oil, IR spectroscopy, rheology

## Abstract

Natural bitumens consist of many molecules whose chemical composition depends on the oilfield and determines the physicochemical properties of the bitumens as materials. Infrared (IR) spectroscopy is the fastest and least expensive method to assess the chemical structure of organic molecules, which makes it attractive in terms of rapid prediction of the properties of natural bitumens based on their composition evaluated in this way. In this work, IR spectra were measured for ten samples of natural bitumens significantly different in properties and origin. Based on the ratios of certain IR absorption bands, bitumens are proposed to be divided into paraffinic, aromatic, and resinous. In addition, the internal relationship between IR spectral characteristics of bitumens, such as polarity, paraffinicity, branchiness, and aromaticity, is shown. A study of phase transitions in bitumens by differential scanning calorimetry was carried out, and the use of a heat flow differential to find hidden points of bitumens’ glass transitions is proposed. Furthermore, the dependences of the total melting enthalpy of crystallizable paraffinic compounds on the aromaticity and branchiness of bitumens are demonstrated. A detailed study of bitumens’ rheology in a wide temperature range was carried out, and characteristic features of rheological behavior for different bitumen classes are revealed. Based on the viscous properties of bitumens, their glass transition points were found and compared with the calorimetric glass transition temperatures and nominal solid–liquid transition points obtained from temperature dependences of bitumens’ storage and loss moduli. The dependences of viscosity, flow activation energy, and glass transition temperature of bitumens on their IR spectral characteristics are shown, which can be used to predict the rheological properties of bitumens.

## 1. Introduction

Bitumens are brown to black solid (or semi-solid at ambient temperature) substances, which are mixtures of heavy hydrocarbons and their derivatives. There are natural and refined bitumens. Natural bitumens are part of fossil fuels and the result of oil deposits’ chemical and biochemical oxidation with the evaporation of lighter oil fractions. The largest reserves of natural bitumens are located in Canada, Venezuela, and Russia [1,2,3,4], where their extraction is carried out mainly by quarrying or mining [5]. In turn, refined bitumens are the residual products of crude oil, coal, and oil shale refining, and are similar in composition and physicochemical properties to natural bitumens.

Bitumens are often described as colloidal dispersions of asphaltenes in an oil phase consisting of saturates (9–18%), aromatics (18–31%), and resins (13–38%), while the content of asphaltenes ranges from 7% to 38% [6,7,8,9,10]. Bitumens are characterized by an amorphous structure and do not have a crystal lattice. As a consequence, their thermophysical properties are described using the softening and glass transition temperatures rather than the melting point. Currently, bitumens are actively used to produce waterproofing construction and roofing materials, but the main area of bitumens’ application is in the road industry, thanks to their specific operational properties providing them with the ability to work in harsh conditions [11]. Mechanical impacts and environmental conditions directly affect the service life of asphalt roads [12,13]. At high temperatures, bitumens soften, and this can lead to rutting, whereas with a decrease in temperature, their brittleness increases, resulting in cracking and gradual destruction of roadways and pavements [14]. Combined effects of high temperatures and long operating times on bitumens cause the loss of volatile compounds [15,16], and this also affects the performance of roadways. Resistance to low-temperature cracking, fatigue cracking, and rutting is largely governed by rheological properties of bitumens and binders on their bases [17], thereby making rheological characterization of bitumens in a wide range of temperatures and mechanical loads is of great importance.

Natural bitumens have a higher viscosity (>10 Pa·s) and a higher density (>1 g/mL) compared to heavy crude oils (>0.1 Pa·s, 0.934–1.000 g/mL) [7,18]. There are no systematic works on a detailed comparative study of the rheology of natural bitumens, but the rheological properties of refined bitumens are similar to those of heavy crude oils. Refined bitumens are capable of exhibiting non-Newtonian behavior, but only at low temperatures and high shear rates [19,20,21,22]. Similarly to crude oils, refined bitumens are characterized by a strong dependence of rheological properties on temperature. At low temperatures, these bitumens are characterized by a solid-like state and brittleness, while an increase in temperature leads them to become viscous liquids [23,24]. Meanwhile, heavy crude oils also turn into a solid-like state when cooled to low temperatures [25]. In other words, refined bitumens and heavy crude oils have qualitatively similar rheological properties that differ only quantitatively. In this regard, the validity of the classifying division of heavy hydrocarbons into two separate classes of heavy crude oils and bitumens is debatable. However, this is correct only for refined bitumens, which are produced from the heaviest residues of crude oils. Natural bitumens may have their own specific hydrocarbon composition, and consequently their rheology may be quite different compared to that of refined bitumens.

The properties of bitumens appear to be largely governed by chemical composition. The high content of petroleum compounds with high either molecular mass or polarity (asphaltenes, long-chain paraffin waxes, resins) determines the basic physicochemical characteristics of bitumens [26,27,28]. Asphaltenes have the greatest influence on the properties of bitumens, increasing their viscosity [29], hardness [30], brittleness, and softening temperature [31]. Over time, polymerization of resins can occur under the influence of oxidative processes together with high temperatures and pressures, leading to an increase in the content of asphaltenes and deterioration of bitumen properties [27,32]. This phenomenon is commonly known as aging. Saturated and aromatic compounds also significantly affect the viscosity and other physicochemical characteristics of bitumens [33], and the crystallizing long-chain paraffins in the form of wax have the greatest influence. The presence of wax causes the brittleness, low plasticity, and poor adhesion of bitumens [34], as well as high viscosity at temperatures below the wax crystallization temperature (30–80 °C) [35].

As a rule, the fractional composition of bitumen (SARA: saturates, aromatics, resins, and asphaltenes) is determined by liquid chromatography [25,36]. Although this method allows obtaining a fairly accurate petroleum composition, it is not quite handy, requiring a relatively long time for analysis, complex and expensive equipment, and toxic eluents. Infrared spectroscopy lacks these disadvantages and can be used as a replacement for liquid chromatography. IR-spectroscopy permits to evaluate SARA composition [37] as well as the relative content of any functional group for a more detailed analysis of composition and structure of bitumens and their classification [38,39].

**Figure 1:**
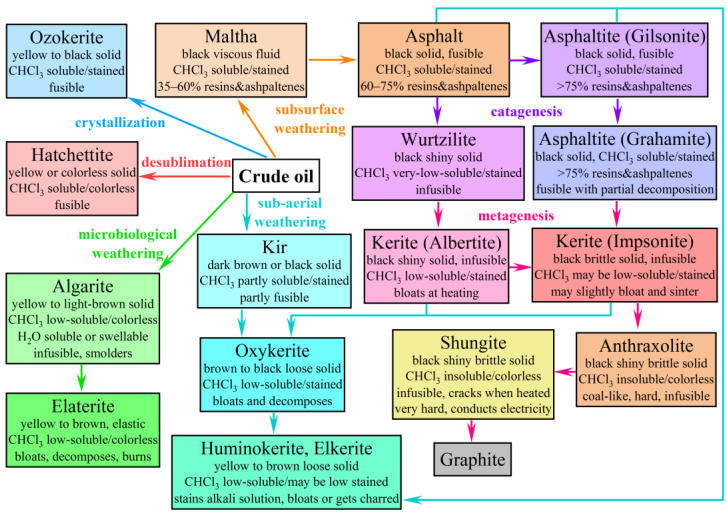
An approximate scheme of relations between the main genetic types of bitumens and ways of their formation and transformation. The inscriptions near the arrows and their colors indicate the bitumen conversion paths, while the rectangles contain names of bitumens, their visual characteristics, and their behavior when heated or in contact with a solvent.

Usually, a classification serves to facilitate the selection of a particular material for some purposes. There are various classifications of natural bitumens as organic minerals according to their genesis, geochemical features, composition, and physicochemical properties [8,40,41,42,43,44,45,46]. These classifications are fairly conditional, and quite often there are situations when bitumens belonging to the same group, e.g., according to their origin, are very different in fractional composition and vice versa. There are different views on the origin of bitumens, which can be formed from kerogen (pre-oil) and crude oil (post-oil) through their transformations [47,48]. In a broad (genetic) definition, bitumens are natural derivatives of crude oils and their pyrogenic analogs generated under high temperatures and tectonic stresses in organic-rich rocks. These bitumens can be classified based on their solubility, fusibility, and formation pathways. Figure 1 shows a generalization of the available information and classifications from the existing works. Bitumens can be formed from crude oils or other bitumens by microbiological (algarites, elaterites), sub-aerial (kirs, oxykerites, huminokerites, elkerites), or subsurface (malthas, asphalts) weathering. These bitumens differ depending on the nature of the weathering, its duration, and the type of parent material. Bitumens can also result from the differentiation of petroleum: crystallization (ozokerites) or desublimation (hatchettites) of its paraffinic compounds. In turn, bitumens (mainly asphalts) can then undergo catagenesis to form asphaltites (in sequence: gilsonites, glance pitches, and grahamites) from the asphaltene-rich feedstock and wurtzilites from the naphthene-rich feedstock. Further, these bitumens may be subjected to metagenesis to form kerites with varying degrees of maturation in sequence: albertites, anchi-, epi-, meso-, and cata-impsonites. Theoretically, further matured kerite may become anthraxolite, subsequently shungite, and finally graphite.

Extremely hard and brittle, infusible, and insoluble bitumens are of limited interest and are very different from their meltable and soluble cousins. This suggests a second narrower (physicochemical) definition of bitumens as natural organic substances soluble in neutral organic liquids under normal conditions. The ability to dissolve often implies the capacity to melt upon heating, and this distinguishes the so-called “true bitumens”: wax-rich ozokerites and hatchettites, and asphaltene-rich malthas, asphalts, and asphaltites (gilsonites). These bitumens do not contain insoluble and non-melting polycondensed compounds with high carbon content—the carboids [49,50]. Further, the scope of practical use of bitumens offers the third narrowest (applied) definition of them as substances with specific technical characteristics, which may have different origins being natural derivatives of crude oil or the product of its refining. When it comes to natural bitumens, asphalts are best suited to this applied definition. In turn, this causes significant confusion in the scientific and technical literature because the words bitumen and asphalt are used synonymously. This is complicated further by using the word “asphalt” to refer to asphalt concrete, which is also known as an asphalt mixture.

The complex nature of bitumens as multi-component systems gives rise to the conditional character of their differentiation, including their distinction from crude oils. By way of example, it is possible to consider the malthas as extra-viscous or extra-heavy crude oils. There is no strict boundary between heavy oils and natural bitumens, and density and viscosity only allow for defining some conventional boundary between them. From the same perspective, the boundary between ozokerites and waxy oils is also conditional. In this case, the value of yielding temperature can be the boundary, considering ozokerites are less fusible. Although the viscosity of petroleum correlates to a certain extent with its density, this dependence is relatively weak [51], and there is no relationship to the melting point in general. An improved way is to predict the viscosity from concentrations of resins and asphaltenes [52], but their content is not always known. At the same time, it can expect that the viscosity correlates with the relative content of some functional groups in bitumen, and then IR spectroscopy can serve to forecast the properties of bitumen. Though rheological properties have an enormous effect on the use of petroleum in various industries, they go ignored when creating classifications of bitumens. Meanwhile, it is an actual task to predict not only the viscosity of crude oils and bitumens at given temperatures but also other features of their complex rheological behavior. Therefore, the present paper is dedicated to the detailed comparative study of IR-spectra and rheological properties of natural bitumens of various origins to generalize and classify this complex group of hydrocarbons. At the same time, since we are dealing with the rheology of bitumens as liquids, only the meltable bitumens will be considered, i.e., the so-called true bitumens: ozokerites, malthas, asphalts, and asphaltites.

## 2. Results and Discussion

### 2.1. Infrared Spectral Characterization

**Figure 2:**
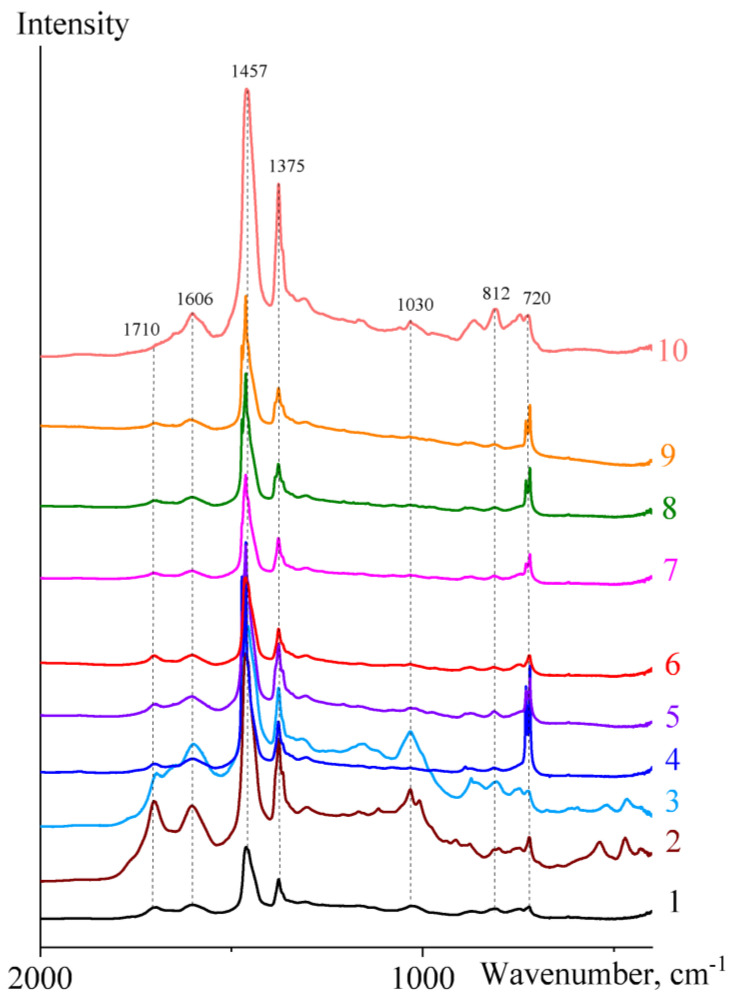
IR spectra of bitumens, whose numbers are indicated in Table 1.

The infrared spectra of bitumens (Table 1) have similarities and differences (Figure 2). All spectra show bands corresponding to chain-like methylene groups (720 cm^−1^), non-substituted aromatic rings (812 cm^−1^), methyl groups (1375 cm^−1^), methyl and methylene groups (1457 cm^−1^), and benzene rings (1606 cm^−1^) [53]. Besides, some bitumens have absorption bands corresponding to sulfoxide (1030 cm^−1^) and carbonyl groups (1710 cm^−1^). Based on the ratios of the maxima of these bands, it is possible to calculate the spectral characteristics of bitumens for their analysis and classification (Table 2).

The ratio of the maxima of the bands corresponding to benzene rings and long-chain methylene groups (*A*_1606_/*A*_720_) characterizes the aromaticity of the samples. It varies in the range of 0.16–2.34. Bitumen **4** has the lowest aromaticity (0.16) and simultaneously contains the lowest concentration of heteroatoms (Table 1). On the contrary, asphaltite **3** contains the highest mass fractions of nitrogen and sulfur. Thus, the content of heteroatoms and aromaticity are interrelated in some way. The inverse parameter, paraffinicity, can be obtained as the ratio of the maxima characterizing the contents of methylene and methyl groups to the maximum proportional to the concentration of benzene rings: (*A*_720_ + *A*_1375_)/*A*_1606_. As expected, asphaltite **3** has the lowest paraffinicity (2.11) but the greatest aromaticity (2.34). Bitumens **8**, **9**, and **4** have the highest paraffinicity (10.6, 10.1, and 9.25, respectively) and the lowest aromaticity (0.16–0.18). In this respect, the parameters aromaticity and paraffinicity are opposite and interchangeable.

**Figure 3:**
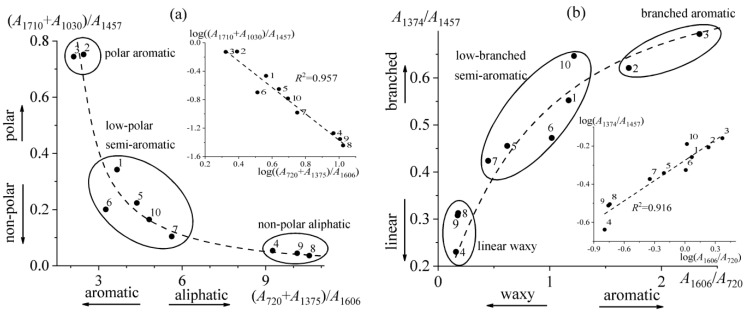
Mutual dependences of bitumen spectral characteristics: (**a**) polarity vs. paraffinicity and (**b**) branchiness vs. aromaticity. Bitumen numbers are indicated near the experimental points. The insets show the same dependencies in logarithmic coordinates.

The branchiness, i.e., the ratio of the relative concentration of methyl groups to that of methyl and methylene groups (*A*_1375_/*A*_1457_), allows estimating the nominal branching degree of alkane fragments for bitumen compounds. The least aromatic bitumen **4** has the lowest branchiness (0.23), while the most aromatic sample **3** has the greatest one (0.69), i.e., the more paraffinic samples are simultaneously less branched. Another spectral parameter of bitumens is the non-substitution of their aromatic compounds (*A*_812_/*A*_1606_). Since this parameter may indirectly characterize the relative content of light aromatics, they are more inherent to bitumen **10** (1.10) and less to bitumen **6** (0.39). Bitumens can also be characterized by relative polarity: the ratio of the sum of the spectral maxima of sulfoxide and carbonyl groups to the spectral maximum of methyl and methylene groups, (*A*_1710_ + *A*_1030_)/*A*_1457_. The least polar are bitumens **4**, **8**, and **9** (0.04–0.05), which also have the highest paraffinicity and the lowest aromaticity. Similarly, the most aromatic bitumens **2** and **3** have the highest polarity (0.73–0.75). Thus, the spectral parameters intercorrelate, which can be considered in more detail using the corresponding graphical dependencies (Figure 3).

Polarity correlates with paraffinicity; higher paraffinicity gives lower polarity (Figure 3a). Based on this, it can conclude that the polar groups of bitumen compounds are mainly associated with their aromatic fragments rather than with aliphatic ones. As a result, two extreme groups of bitumens can be distinguished: polar aromatic (**2** and **3**) and non-polar aliphatic (**4**, **8**, and **9**) bitumens, while the remaining bitumens form an intermediate group of low-polar semi-aromatic samples. It should be noted that the dependence of polarity on paraffinicity is linearized in logarithmic coordinates (the inset in Figure 3a), clearly proving their intercorrelation.

In a similar but unexpected way, branchiness turns out to be interrelated with aromaticity (Figure 3b): higher branchiness gives higher aromaticity. Thus, saturated bitumen compounds have a predominantly linear structure (they are paraffin waxes), whereas aliphatic substituents at aromatic fragments of bitumen compounds have, on the contrary, branched structures. The dependence of branchiness on aromaticity is linear in logarithmic coordinates, which proves the internal relationship of these spectral parameters (the inset in Figure 3b). As in the previous case, the same two groups of bitumens are formed at opposite ends of the dependence: branched aromatic bitumens **2** and **3** and linear waxy bitumens **4**, **8**, and **9**. Bitumens with intermediate values of spectral characteristics can be called low-branched semi-aromatic ones.

The generalization of polarity/paraffinicity and branchiness/aromaticity indicators makes it possible to classify bitumen by a key characteristic feature. Bitumens can differ in the type of components that primarily determine their rheological and other physicochemical properties. These can be crystallizing paraffins that give solid properties, resins/asphaltenes that induce glass transition, or aromatic compounds that provide a relatively high flowability. It can expect that the most polar aromatic bitumens with a branched structure of molecules (samples **2** and **3**) contain a high amount of resin-asphaltene compounds (and this is indeed the case, see Table 1) and therefore can be categorized as resinous. Non- or low-polar bitumens (samples **4**, **5**, and **7**–**9**) with a high content of linear or low-branched crystallizable paraffins can be classed as paraffinic. In turn, the remaining low-polar low-branched semi-aromatic bitumens **1**, **6**, and **10** can be attributed to the group of aromatic bitumens, which are less polar in comparison to the more aromatic resinous bitumens **2** and **3**. In this respect, semi-aromaticity means non-condensed (light) aromatics, while high aromaticity indicates a high content of polyaromatic compounds.

### 2.2. Thermophysical Properties of Bitumens

**Figure 4:**
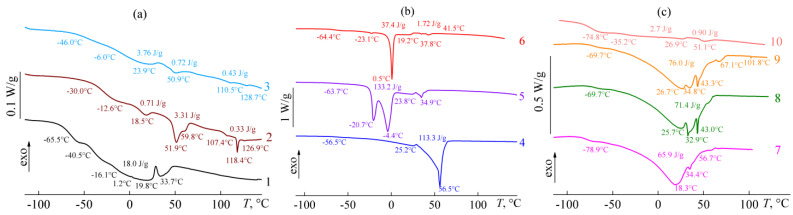
DSC thermograms of bitumens, whose numbers, transition temperatures, and transition enthalpies are shown near the curves. Transition temperatures were found by differentiating the curves.

Thermograms of bitumens differ from each other even more significantly than their IR spectra (Figure 4). Resinous bitumens **2** and **3** have several weakly expressed endothermic transitions in the temperature range from 10 °C to 120 °C, which are probably associated with the melting of paraffin wax impurities, including those of a high molecular nature. At the same time, the glass-transition zone can be highlighted in the low-temperature region for these samples with the broad temperature range due to bitumens’ multi-component compositions. Because of this broadness, it is extremely difficult to determine the exact value of glass transition temperatures from these thermograms.

**Figure 5:**
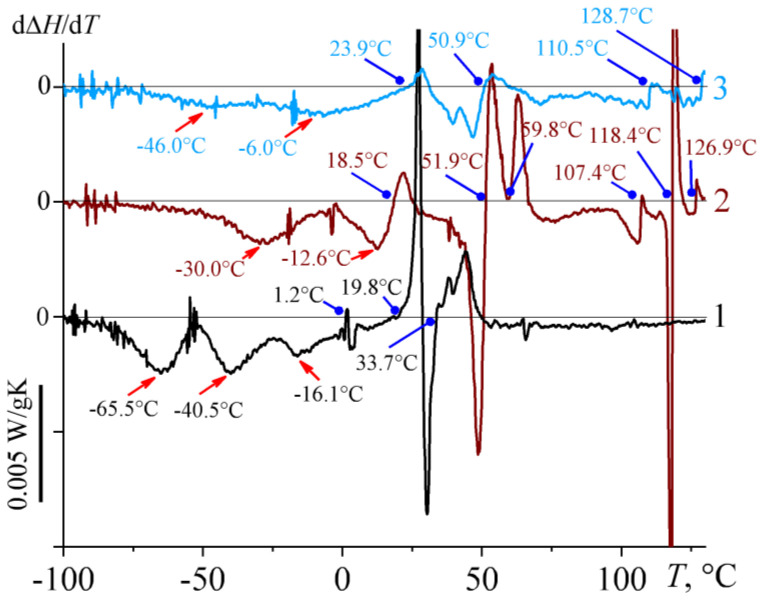
Temperature dependences of differential heat flow for bitumens **1**–**3** as typical examples. The red triangular-ending arrows indicate local minima corresponding to glass transition temperatures, while the blue round-ending arrows mark points at which the rising curves take on zero values, specifying melting temperatures.

However, the glass transition temperature corresponds to the inflection point on the DSC curves, which can be used since the first derivative of some function takes a local minimum or maximum in its inflection point. In our case, this is the point of local minimum since the glass-to-fluid transition at heating reduces the heat capacity of the system and the inflection point is a falling one. Indeed, if the heat flow curves are differentiated with respect to temperature, then the clear minima corresponding to the points of glass transitions can be found on the obtained dependences (Figure 5, shown by red arrows with triangular ends). For bitumen **2**, glass transition occurs at a temperature of −30 °C, whereas bitumen **3** has indication of two glass transition points at −47 °C and −6 °C, which are impossible to detect on thermograms in traditional coordinates. In addition, differential heat flow thermograms make it possible to determine the melting points of paraffin waxes and other compounds more accurately, as heat flow minima appear on differential curves as points at which their rising derivatives take zero (the points are shown by blue arrows with round ends in Figure 5). By way of example, bitumen **2** has six melting points of its constituents. In this case, most of the compounds melt at 51.9 °C, which is the typical melting point of paraffin wax [54]. Bitumen **3** has fewer melting points but is characterized by a comparable amount of paraffin wax, judging by the enthalpy of its melting. However, this wax is more fusible since it melts mainly at 23.9 °C.

Despite the presence of paraffin waxes in bitumens **2** and **3**, their content is negligible. This becomes obvious when considering thermograms of paraffinic bitumens **4**, **5**, and **7**–**9**: the thermal effect of paraffin melting in these bitumens is 66–133 J/g (Figure 4b,c). Since the melting enthalpy of typical paraffin wax is about 210 J/g [55], the concentration of crystallizable paraffins in these bitumens is approximately 31–63%. At the same time, these paraffins differ significantly in melting temperature. Bitumen **4** contains the most hardly fusible paraffins with a melting point of 56.5 °C (Figure 4b). The melting point of paraffins in samples **7**–**9** is lower and equal to 18–35 °C (Figure 4c), suggesting that these paraffins have a lower molecular weight or/and more branched structure. Bitumen **5** contains even easily fusible paraffin compounds with melting temperatures of −20.7 °C and −4.4 °C (Figure 4b), which approximately correspond to the melting temperatures of un-, do-, and tri-decane isomers. In addition, paraffinic bitumens have lower glass transition temperatures compared to resinous bitumens (from −56.5 °C to −78.9 °C vs. from −6 °C to −46 °C), and this can associate with a higher content of non-polar compounds in them—paraffins.

Compared with resinous and paraffinic bitumens, aromatic bitumens **1**, **6**, and **10** have an intermediate total thermal effect of paraffin melting, reaching a maximum of 39.1 J/g in the case of bitumen **6** (Figure 4b). At the same time, paraffins of bitumens **1** and **10** melt within very wide temperature ranges, while paraffins of bitumen **6** melt predominantly at a single temperature of 0.5 °C. The glass transition temperature of aromatic bitumens varies from −16.1 °C to −74.8 °C, which also makes them intermediate between paraffinic and resinous bitumens by the value of this parameter.

**Figure 6:**
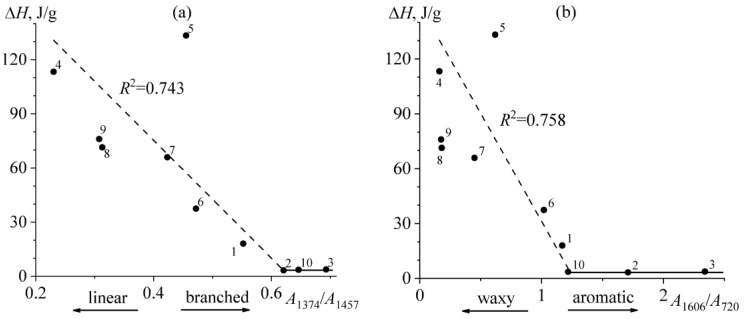
Dependences of the total melting enthalpy of crystallized substances of bitumens on their branchiness (**a**) and aromaticity (**b**). Bitumen numbers are indicated near the experimental points.

Thus, in the series from paraffinic to aromatic and then to resinous bitumen, the temperature of bitumens’ glass transition increases while the content of paraffin decreases. The latter is well illustrated by correlation dependences connecting the total enthalpy of paraffin melting and spectral characteristics of bitumen. The highest correlation is found for the enthalpy of paraffin melting and the branchiness of bitumen (Figure 6a), i.e., the higher branchiness of bitumen compounds leads to a lower content of crystallizing substances in it. Meanwhile, the content of crystallizing substances attains an approximately constant level when a certain branchiness is reached, which is characteristic of resinous bitumens **2** and **3** and aromatic bitumen **10**. Similarly, the value of the total enthalpy of paraffin melting is affected by aromaticity, whose increase causes a decrease of crystallized paraffin content almost to zero (Figure 6b). Other spectral characteristics of bitumen do not correlate meaningfully with the content of crystallizing paraffins (coefficient of determination *R*^2^ < 0.5).

### 2.3. Flow Behavior of Bitumens

**Figure 7:**
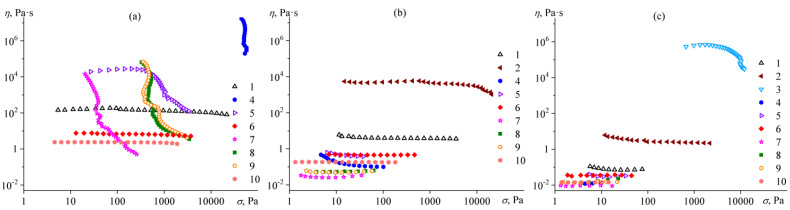
Dependences of effective viscosity on shear stress for bitumens at 25 °C (**a**), 60 °C (**b**), and 135 °C (**c**). Bitumen numbers are indicated in the legends.

Bitumens can be both viscous liquids and solids since their aggregate state and physicochemical properties strongly depend on environmental conditions, particularly temperature. Let us consider the viscosity characteristics of the samples in more detail at three temperatures: 25 °C, 60 °C, and 135 °C (Figure 7).

Even at 25 °C, all aromatic bitumens **1**, **6**, and **10** behave similarly to Newtonian liquids, i.e., their effective viscosity is independent of shear stress (Figure 7a). Resinous bitumens **2** and **3** are glassy substances, which makes it impossible to measure their flow curves at this temperature. In turn, paraffinic bitumens **4**, **5**, and **7**–**9** exhibit non-Newtonian behavior at 25 °C since their effective viscosity decreases with increasing shear stress. At the same time, the non-Newtonian behavior of paraffinic bitumens differs. Bitumens **7**–**9** are characterized by the presence of a yield stress: at shear stresses below the yield stress, these bitumens do not flow, whereas their effective viscosity decreases sharply at higher shear stresses with a tendency to reach a constant value. This yield stress behavior is associated with the presence of solid paraffin particles in bitumens and their interaction with each other to form a structural network [56] whose strength is equal to the yield stress [57]. At stresses above the yield stress, the structural network of paraffin particles is destroyed, accompanied by a decrease in the effective viscosity of paraffinic bitumens to an approximately constant level. At the same time, the effective viscosity of bitumen **4** does not reach a constant level at high shear stresses, probably because these stresses cause wall slipping of the sample instead of destroying its paraffin structure. This phenomenon is probably due to the very high content of high-melting paraffin wax in this bitumen (see Figure 4b).

On the contrary, paraffinic bitumen **5** contains a lot of predominantly low-melting wax, which dissolves in bitumen medium at 25 °C: as a result, this bitumen is capable of flowing with a constant viscosity even at low shear stresses. Nevertheless, the effective viscosity of bitumen **5** also decreases at higher shear stresses, which can be attributed to the destruction of agglomerates of high-melting paraffin wax particles present in this bitumen and having a melting point of 34.9 °C (see Figure 4b).

With an increase of temperature to 60 °C (Figure 7b), almost all paraffinic bitumens become Newtonian liquids (like aromatic bitumens) due to the melting of paraffin wax and its dissolution in a continuous bitumen medium. The exception is bitumen **4**, which contains paraffins with the highest melting temperatures of 28–65 °C (see Figure 4b). As a result, the effective viscosity of this bitumen decreases slightly in the region of low shear stresses at 60 °C, which can attribute to the destruction of agglomerates from unmelted paraffin particles. In addition, resinous bitumen **2** acquires fluidity at 60 °C, which makes it possible to estimate its flow behavior. The viscosity of this bitumen is almost constant in the region of low to moderate shear stresses but decreases slightly at high shear stresses. The latter can associate with the mechanical glass transition of this sample at high shear stresses and rates due to its comparatively high glass transition temperature (−12.6 °C according to DSC data, see Figure 4a). At the same time, resinous bitumen **3** does not acquire fluidity at 60 °C for its testing, although its glass transition temperature is −6 °C according to the DSC. In other words, the viscosity of this bitumen exceeds at least 10^6^ Pa·s at 60 °C, and more significant heating of this sample is necessary to convert it into a fluid form.

An increase in temperature to 135 °C makes it possible to measure the viscosity properties of resinous bitumen **3** exhibiting non-Newtonian behavior since its effective viscosity decreases at high shear stresses (Figure 7c). As in the case of resinous bitumen **2** at 60 °C, the non-Newtonian behavior can explain by mechanical glass transition occurring at high shear rates. Similarly, the effective viscosity of polymer melts and concentrated asphaltene solutions decreases at high shear rates due to their forced transition to a rubbery or glassy state [58,59,60]. At the same time, the non-Newtonian behavior of resinous bitumen **2** disappears at 135 °C because the test temperature becomes significantly higher than its glass transition temperature. All other paraffinic and aromatic bitumens are Newtonian liquids at this temperature. Moreover, their viscosity is below 3 Pa·s, i.e., they, as well as bitumen **2** (2.5 Pa·s), can be considered as road bitumens capable of wetting and mixing with mineral aggregates used to create pavements [61,62], unlike resinous bitumen **3**, where viscosity is too high.

**Figure 8:**
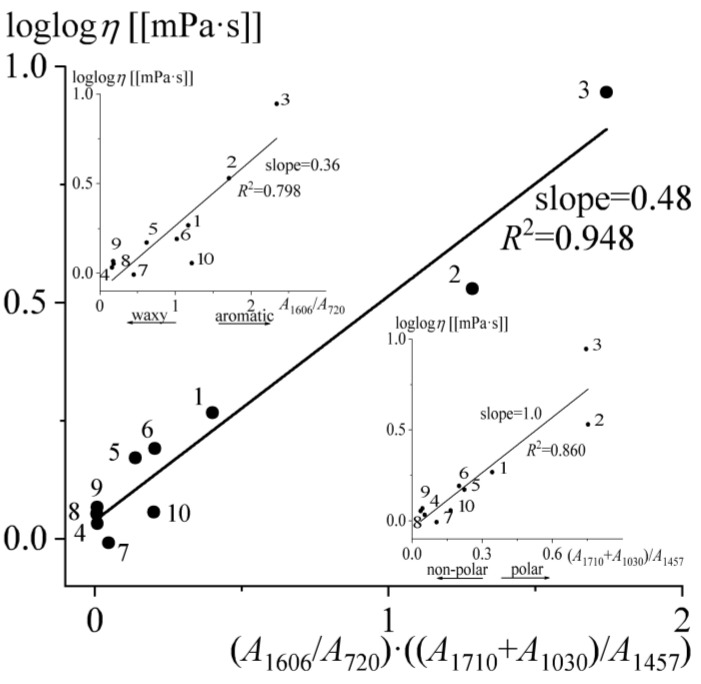
Dependence of the double logarithm of bitumens’ viscosity at 135 °C on the product of aromaticity and polarity. The insets show the same data depending on the aromaticity or polarity of the bitumens.

If considering the dependences of bitumen viscosity on spectral parameters, linear-like dependences can be obtained only by using the double logarithm of bitumen viscosity at 135 °C and expressed in mPa·s (to be able to calculate loglog*η* for all samples). In this case, the best correlations are observed for the dependences of loglog*η* on the polarity (*R*^2^ = 0.860) and aromaticity (*R*^2^ = 0.798) of bitumens (the insets in Figure 8). Moreover, if we combine both spectral parameters in the form of their product, we get a linear dependence of the double logarithm of viscosity with the highest coefficient of determination (*R*^2^ = 0.948, the central part of Figure 8). Thus, the higher the polarity and aromaticity of bitumen, the higher its viscosity. In this case, polarity has a higher effect on the viscosity value than aromaticity, as the dependence of loglog*η* on this parameter has a greater slope (1.0 vs. 0.36, see the insets of Figure 8). This is quite expectable since the higher the polarity of the compounds, the greater the energy of their intermolecular interactions. The same applies to aromaticity since aromatic compounds have stronger dispersion interactions than aliphatic compounds (e.g., aromatic hydrocarbons have a higher solubility parameter than saturated ones, 20.1 MPa^0.5^ vs. 16.1 MPa^0.5^ [63,64]).

### 2.4. Viscoelasticity of Bitumens

**Figure 9:**
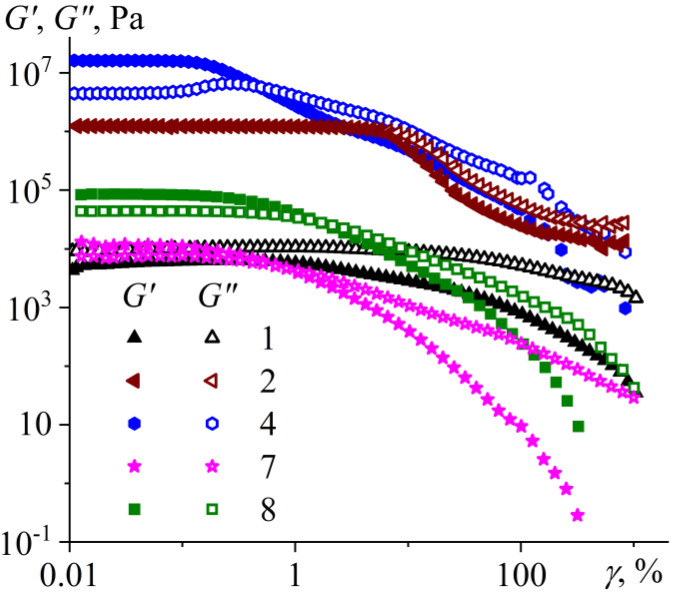
Typical dependences of storage (*G*′) and loss (*G″*) moduli on strain amplitude (*γ*) for bitumens at 25 °C.

Let us consider the viscoelastic characteristics of bitumens and their change with varying deformation conditions (Figure 9). The storage modulus (*G*′) and loss modulus (*G″*) of bitumens do not depend on the amplitude of strain (*γ*) at its small value. In this area of linear viscoelasticity, the samples are deformed without changing their internal structure, which starts to collapse only when some critical strain is reached. Interestingly, the bitumen types differ in the characteristic value of this critical strain.

The structure of paraffin bitumens is the most fragile and is destroyed even at a strain of 0.08–0.13%, which is expressed primarily in a decrease in the storage modulus. Likely, the structural network of paraffin crystalline particles is destroyed at these strain values, as the structural network from solid particles in suspensions usually collapses at a similar strain value [65]. At the same time, the storage modulus of paraffinic bitumens exceeds their loss modulus at low strains, i.e., these bitumens exhibit predominantly solid-like behavior. However, both moduli decrease at strains above the critical value, and the storage modulus drops more rapidly. As a result, the loss modulus starts to exceed the storage modulus at a strain value of approximately 0.4–1.6%, causing liquid-like behavior. In this case, bitumen **4**, which has the highest concentration of crystalline paraffins at 25 °C (according to DSC data, see Figure 4b), has the most fragile structural network. In addition, its loss modulus passes through a local maximum when the strain amplitude increases, i.e., there is a weak strain overshoot with a change in some parameters of the structural network before its destruction [66]. Thus, an increase in the strain amplitude destroys the structural network from paraffin particles and causes the resultant transition of paraffinic bitumens from solid-like to liquid-like behavior.

For resinous bitumen **2**, both moduli are mutually comparable in a wide range of strain amplitudes, and their drop with the transition to the liquid-like state (when *G″* > *G*′) occurs at a much higher strain amplitude of about 5.5%. At this large deformation, perhaps there is a destruction or change in the structure of clusters formed by asphaltenes and resins according to the Yen–Mullins model [67]. Aromatic bitumens have much smaller moduli values, and their loss modulus exceeds the storage modulus even at small deformations, i.e., these bitumens behave like liquids at any strain amplitudes at a given angular frequency. At the same time, a decrease in the storage modulus of these bitumens takes place at a strain of about 0.5%, possibly due to the destruction of agglomerates from crystalline paraffinic impurities present in these samples.

**Figure 10:**
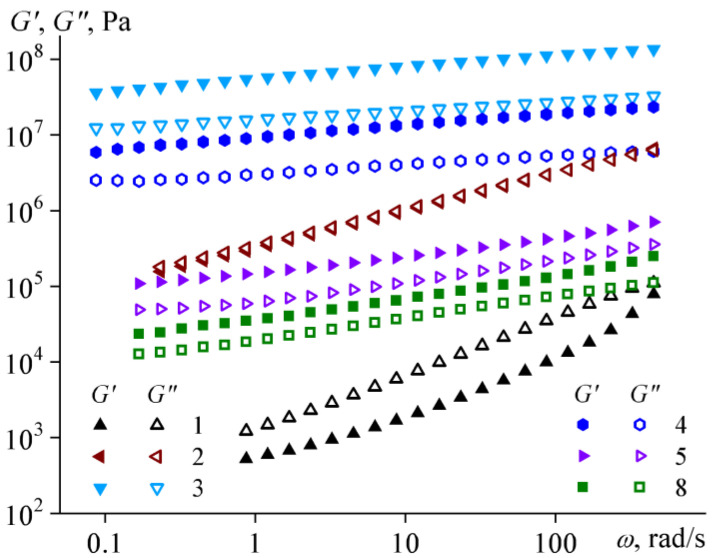
Typical dependences of storage (*G*′) and loss (*G″*) moduli on angular frequency (*ω*) for bitumens at 25 °C.

The storage and loss moduli of bitumens in the linear region of their viscoelasticity depend on the angular frequency *ω* of applied deformation (Figure 10). For aromatic bitumens (e.g., bitumen **1**, the behavior of bitumens **6** and **10** is qualitatively similar), their moduli depend on frequency most strongly. Their loss modulus exceeds the storage modulus in the entire frequency range under consideration, indicating a liquid-like behavior. At the same time, the viscoelastic behavior of these bitumens does not correspond to the Maxwell model, according to which *G*′ ~ *ω*^2.0^ and *G″* ~ ω^1.0^ are at low strain frequencies [68]. In our case, the exponents for aromatic bitumens are much lower (*G*′ ~ *ω*^0.7–1.4^ and *G″* ~ *ω*^0.8–1.0^), probably due to multicomponent compositions, hence a broad relaxation time spectra of bitumens.

Resinous bitumen **2** has storage and loss moduli that coincide in magnitude over the entire frequency range, and their values monotonically grow from 0.2 MPa to 6.6 MPa with increasing frequency (Figure 10). In this regard, this bitumen undergoes mechanical glass transition, which becomes more expressed with increasing frequency. On the contrary, resinous bitumen **3** is already in a glassy state at 25 °C without any additional mechanical action: its moduli change little with variation in the angular frequency, and the storage modulus exceeds the loss modulus, i.e., bitumen **3** demonstrates solid-like behavior [69]. Interestingly, the solid-like behavior is also characteristic of paraffinic bitumens **4**, **5,** and **8**. However, the storage modulus of these bitumens does not significantly exceed the loss modulus, and both moduli are smaller in magnitude than those of glass-formed bitumen **3**. In this respect, paraffinic bitumens behave like gels [70] whose structural network is made from solid paraffin particles. Bitumen **4** contains more paraffins in the crystalline state at 25 °C (see Figure 4b); as a result, its moduli are higher than those of other paraffinic bitumens **5** and **8** but still do not reach the values inherent to glassy bitumen **3**.

Thus, from the viewpoint of the manifestation of a set of rheological properties, resinous bitumens are similar to refined bitumens in their viscoelasticity, glass-formation, and high-shear shear-thinning behavior [7,23,62,71,72]. The yield stress and gel-like behaviors of paraffinic bitumens make them most akin to waxy oils [73,74,75,76], while aromatic bitumens exhibiting Newtonian behavior resemble high-viscous heavy oils [77,78,79].

### 2.5. Rheological Manifestations and the Nature of Temperature Transitions in Bitumens

**Figure 11:**
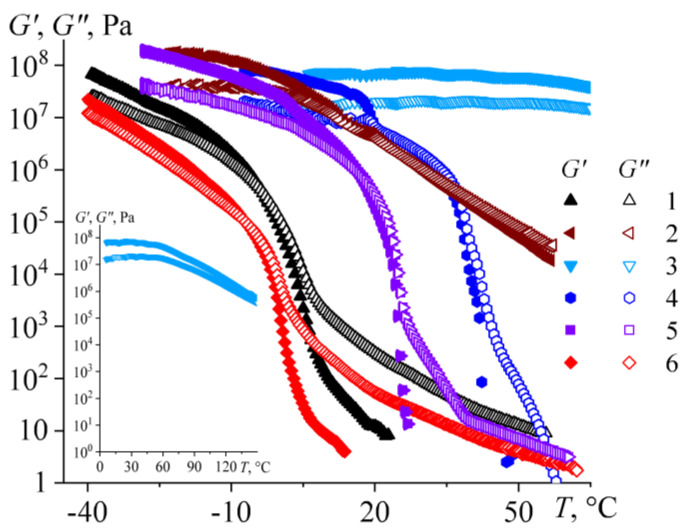
Typical temperature dependences of storage and loss moduli for different types of bitumens, whose numbers are presented in the legend. The inset shows the dependence for bitumen **3** in a broader temperature range.

An increase in temperature leads to a decrease in the storage and loss moduli of bitumens (Figure 11). The moduli of resinous bitumens **2** and **3** change slightly, especially for bitumen **3**. This change is due to the gradual transition of bitumens from a glassy to a fluid state. On the contrary, the moduli of paraffinic bitumens (samples **4** and **5**) drop very strongly in a narrow temperature range, and their storage modulus takes on a zero value in the end. This transition is most likely associated with the melting of paraffin waxes and their dissolution in a bitumen medium. As a result, the structural network that gave elasticity to these bitumens disappears, and they become liquids. The opposite is also true: if liquid-like aromatic bitumens (e.g., **1** and **6**) are cooled deeply, they will acquire elasticity and pass into a solid-like state with the storage modulus exceeding the loss modulus. The cooling of these bitumens causes the crystallization of their low-melting paraffin compounds (see Figure 4), whose particles become interconnected and structured. However, this cannot be considered a positive point, as the crystallization of paraffin compounds causes a substantial rise in the stiffness of bitumen and hence an increase in its brittleness.

The temperature dependences of the storage and loss moduli make it possible to determine the nominal solid–liquid transition point as the temperature of equality between the storage and loss moduli [17,80]. This temperature varies widely from −63 °C for the lowest-viscosity aromatic bitumen **10** to 179 °C for the glassy resinous bitumen **3** (*T_G_*_′=*G″*_, Table 3). The transition from a liquid to a solid state can be associated with both the crystallization of paraffin compounds and/or the glass transition of a continuous bitumen medium. In this respect, the increase in the storage and loss moduli of the bitumens during their cooling (Figure 10) can partially relate to their gradual glass transition. DSC makes it possible to determine the glass transition temperatures of bitumens, but the problem is the presence of several glass transition points for several bitumens (see Figure 4 and Figure 5). It is unclear which of the glass transition temperatures determines the rheological properties of bitumens and how strong its effect is. At the same time, an average glass transition temperature can be estimated rheologically using a temperature dependence of the effective viscosity of a glass-forming liquid and the Williams–Landel–Ferry (WLF) equation [81]:

(1) $$\log\eta =\log\eta_{g}+\frac{-c_{1\;}\left(T-T_{g}\right)\;\;}{c_{2}+\left(T-T_{g}\right)\;\;},$$

where *T_g_* is the average glass transition temperature, *η_g_* is the effective viscosity at the glass transition point, and *c*_1_ and *c*_2_ are the fitting parameters equal to 17.44 and 51.6, respectively.

**Figure 12:**
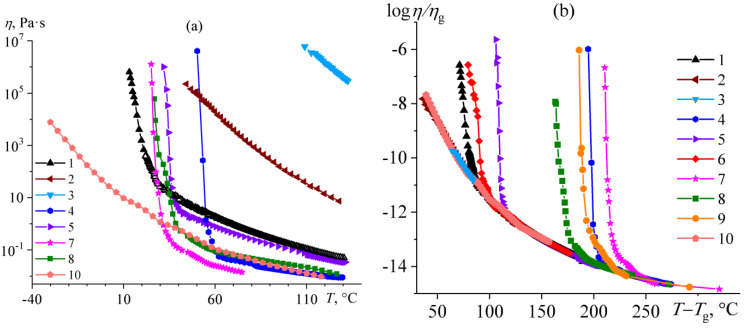
Typical temperature dependences of effective viscosity for bitumens (**a**) and the same dependences in the coordinates of the Williams–Landel–Ferry equation (**b**).

The temperature dependences of the bitumens’ effective viscosity are shown in Figure 12. The viscosity of resinous bitumens **2** and **3** increases monotonically with a decrease in temperature, at least until it can measure. In the case of paraffinic bitumens, a monotonous increase in viscosity occurs only at high temperatures, whereas the reaching of the crystallization point of paraffin compounds during gradual cooling causes a sharp rise in it and then a loss of bitumens’ fluidity. For example, the viscosity of the least-aromatic paraffinic bitumen **4** starts to increase sharply at 60 °C, resulting in the loss of bitumen fluidity at 50 °C. This correlates with the melting point of paraffin compounds of this bitumen at 56.5 °C (Figure 4b). The same is true for other paraffinic bitumens, whose flow loss is associated with the crystallization of their least-fusible paraffins. Moreover, aromatic bitumens **1** and **6** also probably lose fluidity due to the crystallization of their paraffin compounds at temperatures of around 0–10 °C. At the same time, the viscosity of bitumen **10** is characterized by a monotonous increase similar to that of resinous bitumens, which suggests the absence of any structuring of crystalline paraffins, even if they are formed in this bitumen because of deep cooling.

Despite the fundamentally different composition of bitumens, their temperature dependences of viscosity can be reduced to a single dependence using the WLF equation. To do so, the ratio of the measured viscosity to the viscosity at the glass transition point (*η*/*η*_g_) should be a dependent variable, and the difference between the temperature of interest and the glass transition temperature (*T* − *T*_g_) should be an independent variable. The rearrangement of viscosity vs. temperature dependences in these coordinates reveals that the experimental points for all bitumens superimpose perfectly on each other, at least in the region of high temperatures (Figure 12b). These overlapping points correspond to the change in the viscosity of bitumens because of their gradual glass transition due to cooling. However, for all paraffinic bitumens (**4**, **5**, and **7**–**9**) and some aromatic ones (**1** and **6**), the experimental points deviate from a single curve when the temperature decreases. It is the growth in viscosity because of the paraffin crystallization and the formation of a structural network from paraffin particles.

Thus, resinous bitumens lose their fluidity due to glass transition, while paraffinic bitumens lose it due to paraffin crystallization. Aromatic bitumens occupy an intermediate position and can either lose fluidity due to paraffin crystallization or remain liquid down to low temperatures until passing into a glassy state. Despite the different nature of the loss of fluidity by bitumens, the high-temperature section of the temperature dependences of their viscosity makes it possible to estimate the flow activation energy (*E*_A_) using the Arrhenius–Andrade equation [82]

(2) η=AeEART

and to find the glass transition temperature using the WLF equation (*R* is the gas constant, *T* is the absolute temperature, and *A* is the empirical parameter). The glass transition temperature is essential as the point when bitumen conventionally transforms from a high-viscosity liquid to glass, while the flow activation energy characterizes the intensity of the decrease in viscosity of bitumen with increasing temperature. The higher the flow activation energy, the more intense the viscosity decreases upon heating. In this case, the increased flow activation energy is due to either potent intermolecular interactions or branched molecular structure. From a practical point of view, bitumen should have a low flow activation energy, which ensures that its rheological properties remain stable over a wide temperature range, avoiding brittleness when cooling and rutting when heated.

**Figure 13:**
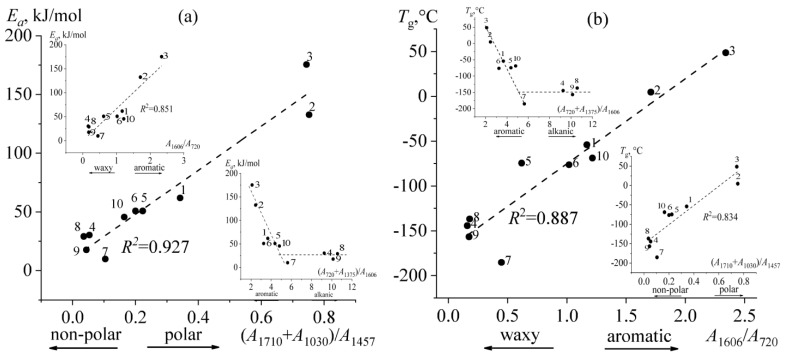
Dependences of (**a**) flow activation energy and (**b**) rheological glass transition temperature on (**a**) polarity and (**b**) aromaticity of bitumens. The insets show the same data but depend on (**a**) the aromaticity and paraffinicity of bitumens or (**b**) their paraffinicity and polarity. Bitumen numbers are indicated near experimental points.

The highest flow activation energy is characteristic of resinous bitumens (Table 3), which can be because they are the most polar of all bitumens (see Table 2, *A*_p_). Indeed, there is a clear correlation between the polarity of bitumen and its flow activation energy with a high coefficient of determination (the central part of Figure 13a, *R*^2^ = 0.927). The flow activation energy also rises with increasing aromaticity of bitumen but is less pronounced (the upper left inset of Figure 13a, *R*^2^ = 0.851). The interaction energy between polar compounds is higher than between non-polar ones, which leads to higher activation energy during the flow of polar media [83]. Likewise, the energy of interactions between aromatic compounds is higher than between aliphatic ones. Thus, the higher the polarity and aromaticity of bitumen, the higher its flow activation energy. This is also confirmed by the fact that the flow activation energy of bitumens decreases with an increase in their paraffinicity but only to some value of this indicator, and then the activation energy reaches an approximately constant level (the lower right inset of Figure 13a).

Similarly, the spectral characteristics of bitumens affect their glass transition temperatures determined rheologically using the WLF equation. In this case, the glass transition temperature correlates slightly better with bitumen aromaticity (*R*^2^ = 0.887, the central part of Figure 13b) than its polarity (*R*^2^ = 0.834, the lower right inset of Figure 13b). As in the case of the flow activation energy, the reason for the increase in the glass transition temperature remains the same: the higher the energy of intermolecular interactions, the higher the glass transition temperature. Identically, an increase in the paraffinicity of bitumen leads to a drop in its glass transition temperature but down to some limit with reaching a constant value (the upper left inset of Figure 13b). Likely, the limited decline in the glass transition temperature is because saturated compounds of bitumens having high paraffinicity are in a crystal state and do not affect the glass transition temperature in its continuous phase. In turn, it may indicate that saturated compounds of paraffinic bitumen melt at high temperatures but incompletely dissolve in a continuous bitumen medium. This conclusion results from the fact that the flow activation energy, measured at high temperatures above the melting point of crystalline paraffin waxes, also becomes approximately constant with increasing paraffinicity (see the lower right inset of Figure 13a).

**Figure 14:**
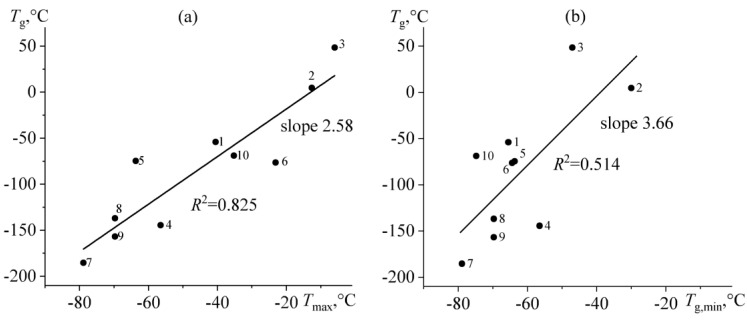
Correlation dependences of the viscometric glass transition temperature on the highest (**a**) or lowest (**b**) glass transition temperatures determined by calorimetry. Bitumen numbers are given near experimental points.

The advantage of the viscometric determination of glass transition temperature is finding a single specific temperature that characterizes the rheological behavior of bitumen. At the same time, according to calorimetry data (Figure 4 and Figure 5), many bitumens have 2–3 glass transition temperatures, which may also not be visible on the thermogram due to a slight change in the heat capacity at glass transition or the imposition of the thermal effect of paraffin melting. Nevertheless, the available data allow for an assessment of the relationship between the glass transition temperatures identified by these two methods (Figure 14).

The glass transition temperature obtained by viscometry correlates more significantly with the highest glass transition temperature determined according to the DSC data (*R*^2^ = 0.825, Figure 14a). There is also a correlation with the lowest glass transition temperature, but it appears weak (*R*^2^ = 0.514, Figure 14b). Therefore, it can be assumed that the highest glass transition temperature of bitumen determines its rheological characteristics when there are several temperatures of pronounced glass transition. At the same time, the viscometric and calorimetric glass transition temperatures interrelate linearly, though not directly proportionally. The viscometric glass transition temperature increases 2.6 or 3.7 times more intensively than the calorimetric maximum or minimum glass transition temperatures, respectively. Thus, the rheological properties of bitumens (e.g., viscosity) are much more sensitive to their composition than their thermophysical characteristics, such as heat capacity that undergoes a stepwise change at the glass transition point.

**Figure 15:**
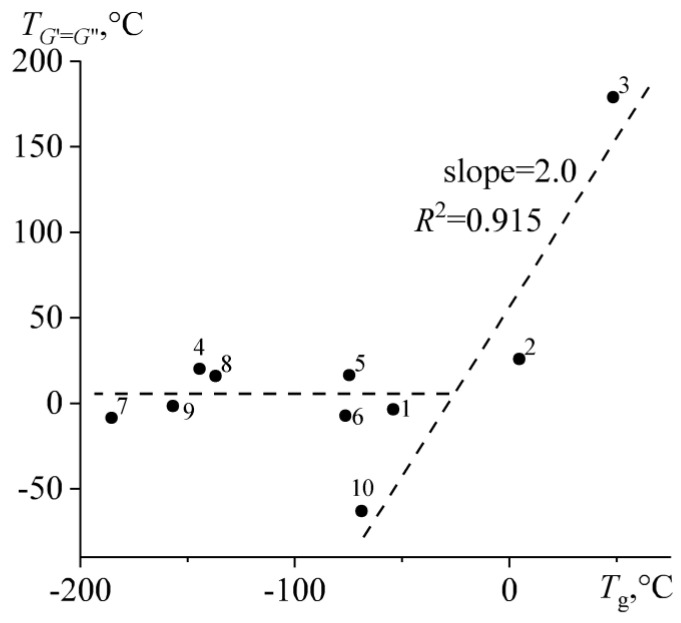
Correlation between the nominal solid–liquid transition temperature and the rheometric glass transition temperature. Bitumen numbers are located near experimental points.

Meanwhile, the nominal solid–liquid transition temperature (when *G*′ = *G″*) does not correlate with the glass transition temperature at first glance (Figure 15). This is because the transition to the solid state of most bitumens is associated with the crystallization of paraffin wax, occurring at a temperature in no way related to the glass transition temperature of the continuous bitumen medium. On the correlation dependence, the data set for these wax-containing bitumens looks like a horizontal section in the zone of low glass transition temperatures. It is also possible to distinguish an inclined section of a straight-line dependence in the zone of large glass transition temperatures but only for resinous bitumens **2** and **3** and aromatic bitumen **10**, which have high glass transition temperatures, the lowest content of crystallizing paraffin compounds (Figure 6), and the highest aromaticity and branchiness (Figure 3b). The nominal solid–liquid transition temperature increases twice as intensely as the glass transition temperature. As a result, both temperatures practically coincide for aromatic bitumen **10**, but for resinous bitumen **3**, the nominal temperature of fluidity loss exceeds the glass transition temperature by 130 °C. Perhaps this phenomenon is because the bitumen with a higher glass transition temperature has a more complex (multicomponent) composition, extending its glass transition over a longer temperature range. In consequence, the storage and loss moduli of bitumen **3** decrease less rapidly with its heating, even when the temperature reaches the glass transition point (see the inset in Figure 11).

## 3. Materials and Methods

### 3.1. Materials

The study was carried out on ten samples of heavy hydrocarbons with significantly different compositions and origin (Table 1). Samples **2** and **4**–**9** are low-sulfur petroleums from Mongolia and China, medium-sulfur samples **1** and **10** are from, respectively, Uzbekistan and Russia, and high-sulfur asphaltite **3** is from the Orenburg region of Russia [84]. Samples **5**, **8**, and **9** originate from different wells of the same Züünbayan oilfield (Mongolia), while **4** and **6** are from two wells of the Fulaerji oilfield (China). At ambient conditions, some of the samples are viscous (**1**, **5**–**7**, **10**) or highly viscous (**8**, **9**) liquids, while some are plastic (**2**) or brittle (**3**) substances. In this respect, all the samples meet the notion of typical bitumens. Meanwhile, the composition of the samples, including the contents of resins (*w*_r_), asphaltenes (*w*_asp_), and paraffin wax (*w*_wax_), varies greatly. The total content of asphaltenes and resins in several samples corresponds to the conventional genetic concept of malthas (**1** and **6**, *w*_r_ + *w*_asp_ ≥ 35%, see Figure 1) or heavier asphalts (**2**, *w*_r_ + *w*_asp_ ≥ 65%) and asphaltites (gilsonites, **3**, *w*_r_ + *w*_asp_ ≥ 75%). Sample **10** has relatively few resins and asphaltenes and hence can be called heavy oil, although it belongs to bitumens in terms of viscosity in the reservoir conditions (*η*_8 °C, 0.4 MPa_ = 24 Pa·s > 10 Pa·s). Other samples (**4**, **5**, **7**–**9**) contain a significant amount of paraffin wax instead of a significant amount of resins and asphaltenes. They have a solid consistency, but only sample **4** melts at temperatures above 50°C (≈61 °C) and can therefore represent ozokerites. Other samples (**5** and **7**–**9**) are more fusible (32–39 °C) and thus can be called waxy oils, although this categorization is only relative. Nevertheless, for the simplicity of presentation, we call all the samples under study bitumens, i.e., bitumens according to their physicochemical definition as natural organic compounds becoming liquids when heated (called true bitumens).

### 3.2. Methods

The elemental composition of bitumens was determined on a Flash 2000 CHNS/O analyzer (Thermo Fisher Scientific, Waltham, MA, USA).

Infrared (IR) spectra were obtained on an IFS 66 v/s spectrometer (Bruker, Germany) in reflection mode using a germanium crystal with an aperture angle of 45° yielding 25 internal reflections. The spectra were recorded with a resolution of 1 cm^−1^ in a wavenumber range from 2000 cm^−1^ to 400 cm^−1^ since that is the wavelength region where specific absorption bands characterizing bitumen samples are located. Based on the ratio of maxima (A) of certain absorption bands, the following spectral characteristics were calculated: aromaticity *A*_1606_/*A*_720_, paraffinicity (*A*_720_ + *A*_1375_)/*A*_1606_, branchiness *A*_1375_/*A*_1457_, and polarity (*A*_1710_ + *A*_1030_)/*A*_1457_ [85,86].

Differential scanning calorimetry (DSC) of samples was performed on a DSC823e calorimeter (Mettler Toledo, USA) in crimped aluminum pans at an argon atmosphere with a flow rate of 70 mL/min and a constant heating rate of 10 °C/min in a temperature range from −100 °C to 150 °C. The calorimeter was calibrated beforehand using n-hexane, distilled water, indium, tin, and bismuth as standard substances. The measurement accuracy was within 0.3 °C and 1 J/g for temperature and enthalpy, respectively [87].

In rheological studies, a rotation stress-controlled rheometer DHR-2 (TA Instruments, USA) was used with a plate–plate measuring unit (the plate diameter: 8 mm, the distance between the plates: 500 μm) for solid-like samples and a cone–plate unit (the plate diameter: 40 mm, the conical surface to plate angle: 2°) for liquid-like ones. Before testing, all samples were kept in an oven at 135 °C for 5 min, then applied to the plate of the measuring unit and cooled to the required temperature. Flow curves of samples were obtained at 25 °C, 60 °C, and 135 °C in a steady-state regime with a step-like change of the shear rate in the range from 10^−3^ s^−1^ to 10^3^ s^−1^; the viscosity measurement time at each shear rate was at least 1 min. The linear viscoelasticity of samples was studied using a constant strain amplitude of 0.1% (at 25 °C) or 10% (at 60 °C) and the angular frequency range 0.0628–628 rad/s. The amplitude dependences of storage and loss moduli were obtained by large-amplitude oscillatory shear at an angular frequency of 10 rad/s and a stepwise increase of strain amplitude from 0.01% to 1000% using the first harmonic of shear stress for calculations [88]. Temperature dependences of viscosity were measured under cooling conditions from 135 °C to −40 °C with a constant shear stress of 100 Pa, while the temperature dependences of storage and loss moduli were obtained in the temperature rise mode from −40 °C to 65 °C with an angular frequency of 10 rad/s, a strain amplitude of 0.1%, and a temperature change rate of 2 °C/min.

## 4. Conclusions

The study of infrared spectra, thermograms, and rheological properties of natural meltable bitumens of various origins and genetic types revealed their following features.

- The dominant constituents of bitumens determine their rheological and thermophysical properties: glass-forming resin-asphaltene compounds, crystallizing paraffin waxes, or aromatics acting as diluents.
- The combination of interrelated infrared spectral characteristics—paraffinicity, aromaticity, polarity, and branchiness—allows for dividing bitumens into three classes: resinous, paraffinic, and aromatic.
- The high glass transition temperature determines the behavior of resinous bitumens (asphalts, gilsonites): they are viscoelastic pseudoplastic glass-forming liquids with a low content of crystallizing paraffin compounds that do not affect bitumens’ rheological properties.
- Crystallizing paraffin compounds give paraffinic bitumens (waxy oils, oxykerites) a gel-like state and a yield stress behavior at low temperatures, disappearing upon heating and melting paraffin crystals.
- Aromatic bitumens (heavy oils, malthas) are Newtonian liquids that can acquire viscoelasticity and non-Newtonian behavior upon cooling due to glass transition or paraffin crystallization.
- The transition of paraffinic and resinous bitumens from a liquid-like to a solid-like state is caused by the paraffin crystallization and the glass-transition of continuous medium, respectively, while the nature of the liquid–solid transition of aromatic bitumens (crystallization or glass-transition) depends on the paraffin content in them.
- The temperature dependence of viscosity is a single universal dependence for bitumens having low paraffinicity or when the test temperature is high, but paraffin crystallization at cooling causes the viscosity deviation from a single dependence.
- An increase in polarity, aromaticity, and branchiness of bitumens (their transition from paraffinic to aromatic and then to resinous) elevates their viscosity, flow activation energy, and glass transition temperature.
- Infrared spectral characteristics allow for predicting such bitumen parameters as melting enthalpy and thus approximate content of crystallizing paraffin compounds, flow activation energy, viscosity, and glass transition temperature.

## Figures and Tables

**Table 1:** Basic characteristics of natural heavy hydrocarbons under study.

#	Oilfield	ρ*, g/mL	C, wt%	H, wt%	S, wt%	N, wt%	H/C, at/at	*w*_r_, wt%	*w*_asp_, wt%	*w*_wax_, wt%	Genetic Type
**1**	Khaudag, Uzbekistan	0.98	82.09	11.24	3.8	0.28	1.64	50.4	9.4	3.9	Maltha
**2**	Bayan-Erkhet, Mongolia	1.03	79.10	10.73	0.47	0.75	1.63	60.2	6.1	1.7	Asphalt
**3**	Ivanovka, Russia	1.12	76.15	8.60	6.07	1.05	1.36	12.7	69.2	0.4	Asphaltite (Gilsonite)
**4**	Fulaerji, China	0.92	86.38	13.32	<0.1	0.17	1.85	34.1	0.2	22.4	Ozokerite
**5**	Züünbayan, Mongolia	0.83	80.77	12.00	<0.1	0.24	1.78	8.5	1.0	26.4	Waxy oil
**6**	Fulaerji, China	0.93	85.57	12.39	<0.1	0.20	1.74	17.9	33.0	16.3	Maltha
**7**	Toson-Ul, Mongolia	0.84	85.34	13.24	<0.1	0.14	1.86	5.7	0.3	24.9	Waxy oil
**8**	Züünbayan, Mongolia	0.90	83.77	13.09	<0.1	0.23	1.88	25.5	0.4	15.7	Waxy oil
**9**	Züünbayan, Mongolia	0.89	85.26	13.34	<0.1	0.24	1.88	14.7	0.2	24.0	Waxy oil
**10**	Ashalcha, Russia	0.96	82.64	11.21	3.9	0.29	1.63	23.8	7.5	0.2	Heavy oil

* *ρ* is the density, C, H, S, and N are the mass fractions of the corresponding atoms, H/C is the atomic ratio of hydrogen and carbon atoms, and *w*_r_, *w*_asp_, and *w*_wax_ are the mass fractions of resins, asphaltenes, and paraffin waxes, respectively (see Section 3.1).

**Table 2:** Spectral characteristics of bitumens presented in Table 1.

#	*A*_a_ *	*A*_w_	*A*_b_	*A*_s_	*A*_p_	Character of Material		
						*A*_p_/*A*_w_	*A*_b_/*A*_a_	Summary
**1**	1.17	3.66	0.55	0.72	0.34	low-polar semi-aromatic	low-branched semi-aromatic	aromatic
**2**	1.71	2.46	0.62	0.44	0.75	polar aromatic	branched aromatic	resinous
**3**	2.34	2.11	0.69	0.54	0.74	polar aromatic	branched aromatic	resinous
**4**	0.16	9.25	0.23	0.47	0.05	non-polar aliphatic	linear waxy	paraffinic
**5**	0.62	4.38	0.46	0.50	0.22	low-polar semi-aromatic	low-branched semi-aromatic	paraffinic
**6**	1.02	3.26	0.47	0.39	0.20	low-polar semi-aromatic	low-branched semi-aromatic	aromatic
**7**	0.45	5.63	0.42	0.63	0.10	low-polar semi-aromatic	low-branched semi-aromatic	paraffinic
**8**	0.18	10.6	0.31	0.72	0.04	non-polar aliphatic	linear waxy	paraffinic
**9**	0.18	10.1	0.31	0.68	0.04	non-polar aliphatic	linear waxy	paraffinic
**10**	1.22	4.82	0.65	1.10	0.16	low-polar semi-aromatic	low-branched semi-aromatic	aromatic

* *A*_a_ is the aromaticity *A*_1606_/*A*_720_, *A*_w_ is the paraffinicity (*A*_720_ + *A*_1375_)/*A*_1606_, *A*_b_ is the branchiness *A*_1375_/*A*_1457_, *A*_s_ is the non-substituted aromaticity *A*_812_/*A*_1606_, and *A*_p_ is the polarity (*A*_1710_ + *A*_1030_)/*A*_1457_.

**Table 3:** Temperature characteristics of bitumens.

#	*T_G_*_′ = *G″*_, °C	*E*_A_, kJ/mol	log*η*/*η*_g_	*T*_g_, °C	*T*_g, min_, °C	*T*_g, max_, °C
**1**	−3.4	61.7	12.39	−54.1	−65.5	−40.5
**2**	26.0	132.6	13.3	4.6	−30.0	−12.6
**3**	179.0	175.4	16.29	48.4	−47.0	−6.0
**4**	20.2	30.2	12.56	−144.4	−56.5	−56.5
**5**	16.5	50.8	12.50	−74.6	−63.7	−63.7
**6**	−7.2	50.8	12.19	−76.4	−64.4	−23.1
**7**	−8.5	9.87	12.78	−185.4	−78.9	−78.9
**8**	16.1	29.0	12.23	−136.9	−69.7	−69.7
**9**	−1.5	17.6	12.93	−156.8	−69.7	−69.7
**10**	−63	45.5	11.56	−68.9	−74.8	−74.8

## Data Availability

The data presented in this study are available upon request from the corresponding author.
